# Selective modulation of local linkages between active transcription and oxidative demethylation activity shapes cardiomyocyte-specific gene-body epigenetic status in mice

**DOI:** 10.1186/s12864-018-4752-4

**Published:** 2018-05-10

**Authors:** Mayumi Oda, Shunichi Wakabayashi, N. Ari Wijetunga, Shinsuke Yuasa, Hirokazu Enomoto, Ruri Kaneda, Sung Han Yoon, Nishant Mittal, Qiang Jing, Masako Suzuki, John M. Greally, Keiichi Fukuda, Shinji Makino

**Affiliations:** 1 Center for Integrated Medical Research, School of Medicine, Keio University, Shinjuku, Tokyo, 160-8582 Japan; 2 Department of Cardiology, School of Medicine, Keio University, Shinjuku, Tokyo, 160-8582 Japan; 3 Systems Medicine, School of Medicine, Keio University, Shinjuku, Tokyo, 160-8582 Japan; 4 Health Center, School of Medicine, Keio University, Shinjuku, Tokyo, 160-8582 Japan; 5 Center for Epigenomics, Albert Einstein College of Medicine, Bronx, NY 10461 USA; 6 Present Address: Systems Medicine, School of Medicine, Keio University, Shinjuku, Tokyo, 160-8582 Japan

**Keywords:** DNA methylation, Gene length, 5-hydroxymethylation, Cardiomyocytes, Epigenome

## Abstract

**Background:**

Cell-type-specific genes exhibit heterogeneity in genomic contexts and may be subject to different epigenetic regulations through different gene transcriptional processes depending on the cell type involved. The gene-body regions (GBRs) of some cardiomyocyte (CM)-specific genes are long and highly hypomethylated in CMs. To explore the cell-type specificities of epigenetic patterns and functions, multiple epigenetic modifications of GBRs were compared among CMs, liver cells and embryonic stem cells (ESCs).

**Results:**

We found that most genes show a moderately negative correlation between transcript levels and gene lengths. As CM-specific genes are generally longer than other cell-type-specific genes, we hypothesized that the gene-body epigenetic features of CMs may support the transcriptional regulation of CM-specific genes. We found gene-body DNA hypomethylation in a CM-specific gene subset co-localized with rare gene-body marks, including RNA polymerase II (Pol II) and p300. Interestingly, 5-hydroxymethylcytosine (5hmC) within the gene body marked cell-type-specific genes at neonatal stages and active gene-body histone mark H3K36 trimethylation declined and overlapped with cell-type-specific gene-body DNA hypomethylation and selective Pol II/p300 accumulation in adulthood. Different combinations of gene-body epigenetic modifications were also observed with genome-wide scale cell-type specificity, revealing the occurrence of dynamic epigenetic rearrangements in GBRs across different cell types.

**Conclusions:**

As 5hmC enrichment proceeded to hypomethylated GBRs, we considered that hypomethylation may not represent a static state but rather an equilibrium state of turnover due to the balance between local methylation linked to transcription and Tet oxidative modification causing demethylation. Accordingly, we conclude that demethylation in CMs can be a used to establish such cell-type-specific epigenetic domains in relation to liver cells. The establishment of cell-type-specific epigenetic control may also change genomic contexts of evolution and may contribute to the development of cell-type-specific transcriptional coordination.

**Electronic supplementary material:**

The online version of this article (10.1186/s12864-018-4752-4) contains supplementary material, which is available to authorized users.

## Background

In mammals, at least one-third of the total length of the genome is transcribed as the pre-mRNA of coding genes, and individual gene-body lengths are highly heterogeneous and can contain a few hundred to a million base pairs. Such variations in genomic size are mostly attributed to the presence of intronic and repetitive sequences [1], differences between different organisms and even between eutherians and metatherians [2]. Interestingly, important cardiomyocyte (CM)-specific genes include several extremely long genes with structures consisting of numerous exons and introns such as those that encode titin (278 kb in mice), the ryanodine receptor (554 kb) and dystrophin (2256 kb) [3]. In addition to structural protein genes, the gene encoding transcription factor Mef2c is also long (163 kb). Such long gene lengths may be disadvantageous during transcription because they require that transcriptional elongation be maintained over long distances without interruption. In addition, these long gene sequences are expressed at equally high levels in CMs in the same nuclear environment where housekeeping (HK) genes are also highly and constitutively transcribed; thus, cell-type-specific epigenetic features that overcome the disadvantages of such long genes must be present.

Epigenetic features alter the functioning of the nucleus on a variety of scales from individual gene units to the entire nucleus [4]. 5-methylcytosine (5mC) is distributed globally in the mammalian genome, covering the vast majority of its CpGs [5], which is characteristic for mammals but which diverges from features of “mosaic DNA methylation”. De novo DNA methyltransferase Dnmt3 contributes to an increase in the DNA methylation rate during development [6]; Dnmt1 and hemimethylated CpG binding protein Np95 maintain a DNA methylation state during most stages of development [7]. In contrast, DNA demethylation occurs “passively” through DNA synthesis or “enzymatically” and “actively” through thymine DNA glycosylase-mediated base excision repair [8]. During active demethylation, 5mC is oxidized by the Tet protein to produce 5-hydroxymethylcytosine (5hmC), 5-formylcytosine (5fC) and 5-carboxycytosine (5caC) [9, 10]. 5fC and 5caC are rapidly replaced with cytosine via thymine DNA glycosylase-mediated base excision repair for demethylation [11, 12], while in most cases 5hmC persists at different levels in different cell types [13]. Interestingly, it has been shown that these oxidative cytosine bases have dynamic and specific binding partners and thus may play an important role as ubiquitous epigenetic substrates [14, 15].

Gene-body regions (GBRs) are sites of transcriptional elongation in the genome. Several studies have demonstrated that active genes are hypermethylated [16]. In embryonic stem cells (ESCs) and differentiated cells, targeted gene-body DNA methylation is established and maintained through the recruitment of Dnmt3b to SET2-dependent H3K36 trimethylation, which is modified through the elongation of RNA polymerase II (Pol II) [17]. However, in muscle cells, there is a negative relationship between gene-body DNA methylation levels and transcript levels [18–20]. In contrast, in neural cells, large accumulations of 5hmC have been observed in the GBRs of active genes [21–23]. Thus, active genes present diverse epigenetic modifications in GBRs, and the relationship between methylation patterns and gene activity remains unknown.

In the genome, HK genes include hypomethylated promoters with conserved CpG-enriched regions known as CpG islands (CGIs) [24]. These characteristics indicate that the cooperation of the genomic context with epigenetic modifications establishes and maintains the stable expression of constitutive genes. In contrast, most cell-type-specific genes include fewer CpG islands in promoter regions than active constitutive genes [25]. In many vertebrates, including more long-lived mammals, the transcriptional state of cell-type-specific genes must be maintained for a long time after birth and requires that high levels of stability are maintained. Given such demands, it is surprising that gene length heterogeneity is increased in vertebrate genomes, which are typically larger and more complex than those of other organisms [26]. Here we demonstrate that the CM-specific DNA methylation epigenome corresponds with the difference between cell-type-specific genes and constitutively expressed genes and that it is likely to regulate different local molecular interactions specific to cell-type-specific genes. Variations in systems of epigenetic expression found between cell types and heterogeneities in genomic components, including those related to gene length diversity levels found in cell-type-specific genes, are also discussed.

## Results

### Relationships between genomic contexts and expression levels differ between cell-type-specific and HK genes

**Fig. 1:**
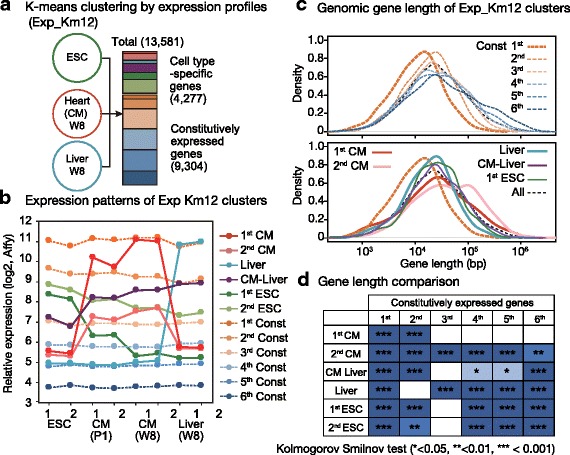
CM-specific genes are longer than constitutively expressed genes with similar expression levels. **a** Gene categorization by expression patterns from all three cell types (Exp_Km12). ESC: embryonic stem cells; CM: cardiomyocytes. W8: adult, 8 weeks old. **b** Relative gene expression patterns of 12 clusters. Const; constitutively expressed gene. P1: neonatal day 1. **c** Gene length distribution among the 12 clusters of CMs and Const genes (top) and among the cell-type-specific genes (bottom). **d** Statistical evaluation of gene lengths among the clusters via Kolmogorov Smilnov testing

Bona fide “cell-type-specific gene” data can be affected by the quality of expression data and/or by the purity of a cell population. In this study, to draw a transcriptional regulation-based comparison between cell-type-specific and constitutively expressed genes, we experimentally categorized gene sets based on the actual expression profiles of cell populations with higher levels of homogeneity, isolated CMs, liver tissues (hepatocytes) and ESCs using the summarized robust multichip average (RMA) values of 3′ expression microarrays [27]. We annotated 13,581 genes according to gene-body DNA methylation levels determined from HELP tagging assays (described below) and we separated these into 12 groups by their expression profiles via K-means clustering (Exp_Km12: Fig. 1a, Additional file 1: Figure S1). Two groups of genes exhibited CM-specific expression patterns and presented different expression levels in CMs, while another two groups exhibited ESC-specific expression patterns. Another group contained liver-specific genes, and one contained genes commonly expressed in both CMs and liver cells; the remaining six groups contained constitutively expressed genes identified in the three cell types with different expression levels (Fig. 1b, Additional file 1: Figure S1). This categorization recognized 68% of the genes as constitutively expressed genes with various expression levels. Previous reports examining the transcriptome of 18 to 47 cell types show that up to 40% of human genes are HK genes [28] consistent with the ratio of active constitutive genes, representing the highest to third highest levels of expression (1st – 3rd Const genes).

Interestingly, when the gene length of the constitutively expressed genes was compared to their expression level, the genes with higher transcript levels tended to be shorter (Fig. 1c). This tendency was observed across different cell types (Additional file 1: Figure S2A). In contrast, despite the fact that the CM- and liver-specific gene groups show high expression levels in addition to the constitutive gene group (1st Const; HK gene), we observed that the two CM-specific gene groups contained genes with a wider and longer length distribution than the highly expressed constitutive gene population (Fig. 1c), which was also confirmed in our transcriptional level-matched comparison (Additional file 1: Figure S2B). Based on the Kolmogorov-Smirnov test, the CM-specific genes (1st CM) were closer in length to the 3rd-6th constitutive gene groups, the liver genes were similar in length to the 2nd constitutive gene group, and comparisons with high expression levels of the cell-type-specific genes (1st CM and Liver group) show similar transcriptional levels to those of the 1st Const gene group in corresponding cell types (Fig. 1d). Among the 12 gene groups, the mean length of the genes was longest for the CM-specific gene group with lower expression levels (2nd CM), whose mean length was longer than that of the 6th constitutive gene group, and this CM-specific gene group displayed similar expression levels to those of the 3rd constitutive gene group (Fig. 1d and Additional file 1: Figure S3A). In summary, the negative correlation found between transcriptional activity and gene length denotes a potential suppression of higher transcription levels of longer genes under the evolutionary precondition that gene lengths are more inhibitory, while CM-specific genes exhibit equivalent transcription levels despite being longer than constitutive genes. Therefore, cell-type-specific gene expression systems may overcome the disadvantages of gene length, and a genome was selected through the survival of affected individuals.

Advantageous features of the genomic structure of constitutive genes were observed in CGIs near the promoter. CGIs are more preserved in active constitutive genes and are depleted from CM-specific gene promoters (Additional file 1: Figure S3B), indicating that the promoter of cell-type-specific genes is less frequently activated and thus may accumulate weaker features for constitutive expression. Taken together, cell-type-specific and constitutive genes might be differentially regulated in their active expression phase via different genomic regulatory elements and epigenetic mechanisms. On this basis, we hypothesized that epigenetic regulation may selectively increase the transcriptional efficiency of cell-type-specific genes that lack advantageous genomic structures.

### The accumulation of oxidative 5mC derivatives leads to gene-body DNA hypomethylation in CM-specific genes.

As CM-specific genes are longer than the expected length predicted from the relationship between the transcriptional levels and gene lengths of constitutive genes, CMs might exhibit cell-type-specific “epigenetic” transcriptional support traits to form their transcriptomes to compensate for the disadvantages of long gene transcription features. Interestingly, hypomethylation has been observed in the GBRs of CM-specific genes [18]. We examined the cell-type specificity and timing of gene-body DNA methylation changes occurring in CMs.

Using HELP tagging assays to examine genome-wide DNA methylation events (Additional file 1: Figure S4), we found that representative CM-specific genes including *Myh6, Myh7, Myl2*, and *Tnnt2* undergo relative hypomethylation in both their promoters and GBRs (Additional file 1: Figure S4A-C) as reported previously [18]. We also found that decreased levels of gene-body DNA methylation accompany the entry into the neonatal stage from the embryonic stage as indicated by increased Angle values and hypomethylation signals (from E9 to P1, Additional file 1: Figure S4A-C). The quantitative features of HELP were validated by PCR-based local bisulfite sequencing and public MethylC-seq data (Additional file 1: Figure S4D-F) [18].

**Fig. 2:**
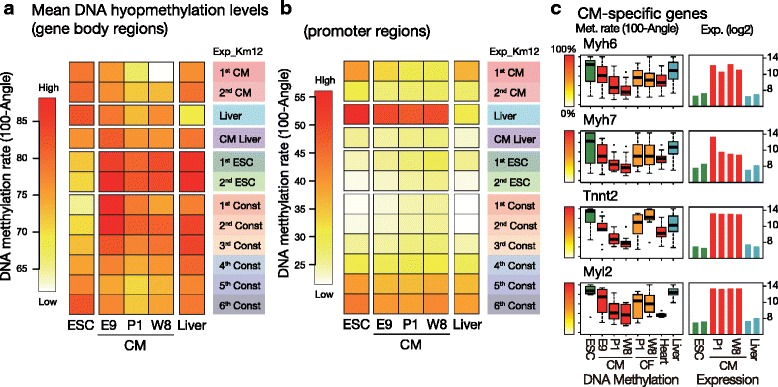
Gene-body hypomethylation was concentrated in the CM-specific gene cluster in CMs. **a**-**b** The mean of gene-body (**a**) and promoter (**b**) DNA methylation levels in the 12 groups in Exp_Km12. (100-HELP Angle) values are shown as approximate DNA methylation values. E9: embryonic day 9. **c** Examples of gene-body DNA methylation comparisons drawn between cardiac genes of multiple cell types. Note that hypomethylation levels in CMs were decreased as cells matured. CF: cardiac fibroblasts

Next, to assess whether the observed gene-body hypomethylation is selectively targeted to cell-type-specific genes, we performed a genome-wide comparison of gene-body and promoter DNA methylation levels of the CMs at different developmental stages. First, the differentiated cell types showed different DNA methylation patterns between the gene-body and promoter regions while ESCs showed a similar pattern between the promoter and GBRs and especially in the constitutive genes (Fig. 2a-b). In terms of the cell-type-specific gene clusters, each cell-type-specific cluster exhibited the lowest DNA methylation levels for each cell type, while the hypomethylation of CM-specific genes in CMs was the lowest among the cell types (Fig. 2a). Over the course of development, the GBRs of embryonic CMs (CM, E9) exhibited higher levels of DNA methylation than those observed in later stages of development, and higher gene-body methylation levels were also observed in active constitutive and ESC-specific genes than those in CM-specific and liver-specific genes. The GBRs of CM-specific genes (1st CM) were increasingly demethylated in the neonatal and adult stages and eventually became the most heavily hypomethylated regions in adult CMs (CM, W8, Fig. 2a), which was also observed in individual CM-specific genes (*My6, Myh7, Tnnt2* and *Myl2*, Fig. 2c). However, such selective changes were not observed in the promoter regions, which were primarily hypomethylated and which remained less altered during CM maturation (Fig. 2b). Thus, this finding indicates that the gene-body DNA methylation profiles of CM-specific genes are dynamically altered during cell maturation and undergo different changes from those found in promoter regions. In contrast, such alterations in DNA methylation levels were not obvious in the GBRs of ESCs (Fig. 2a and b). The gene-body hypomethylated active genes with hypomethylated promoter regions were minor (Additional file 1: Figure S5) and therefore do not significantly affect the correlation between gene-body hypermethylation and active transcription [16].

### Gene-body 5hmC accumulation in CMs distinguishes CM-specific genes from constitutively expressed genes

The cell-type-specific hypomethylated regions described above are considered to result from de novo and specific demethylation occurring during CM maturation (Fig. 2a). Given that active demethylation via the Tet-mediated oxidation pathway generates intermediate products such as 5hmC, we hypothesized that gene-body hypomethylation occurring in cell-type-specific genes may be enriched with active demethylation derivatives. We assessed whether 5hmC accumulation overlaps with hypomethylated GBRs. To investigate the gene population and the timing of 5hmC accumulation in GBRs, a genome-wide analysis of BGT-mediated 5hmC enrichment (fhM-Seal-seq) was performed using heart and liver tissues at the foetal, neonatal and adult stages and ESCs. For the genome-wide comparison, the read frequency per kilobase of transcript per million mapped reads (FPKM) was calculated for the promoter (+/− 2 kb) and GBRs and was normalized (scaled) according to different noise levels measured between the regions.

**Fig. 3:**
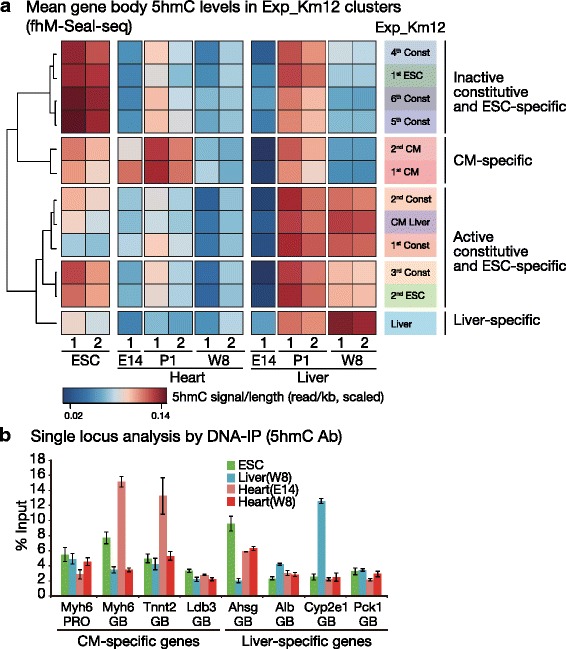
5hmC specifically accumulated in the GBRs of CM-specific genes in the neonatal stage. **a** Gene-body summary of 5hmC signals categorized by Exp_Km12 cluster. E14: embryonic day 14. **b** DNA immunoprecipitation of cardiomyocyte (CM)- and liver-specific genes from ESCs and from embryonic and adult tissues using the 5hmC antibody

The genome-wide comparison shows that 5hmC accumulation was observed in two CM-specific gene clusters (1st and 2nd CM) during the embryonic and neonatal periods, and then this declined in the adult tissue (Fig. 3a), which was validated by local quantitative PCR-based measurement via two different affinity-based methods; antibody-based and chemical bond-based 5hmC DNA enrichment (Fig. 3b and Additional file 1: Figure S6). This transient 5hmC accumulation peak was not observed in the other hypomethylated regions or in inactive constitutive genes, indicating that the active demethylation process distinguishes active from inactive hypomethylated GBRs in CMs (Fig. 3a). In contrast, liver tissues showed broad levels of 5mC turnover in GBRs at the neonatal stage, whereas in the adult tissue 5mC turnover was limited to all active clusters including liver-specific, CM-liver common and active constitutive clusters (Fig. 3a). Thus, gene-body 5mC turnover patterns observed in CMs distinguished the CM-specific genes from the constitutively expressed active genes, whereas they more widely marked active genes in the liver. Interestingly, 5hmC in ESC does not mark active genes, as both ESC-specific and inactive constitutive genes were marked. Taken together, 5hmC forges various linkages depending cell types and developmental stages involved. In mature differentiated cells, 5hmC acts as a roughly active mark, though preferences are dependent on the cell types involved.

### Active epigenetic molecular marks expanded to GBRs with cell-type-specific 5mC turnover

**Fig. 4:**
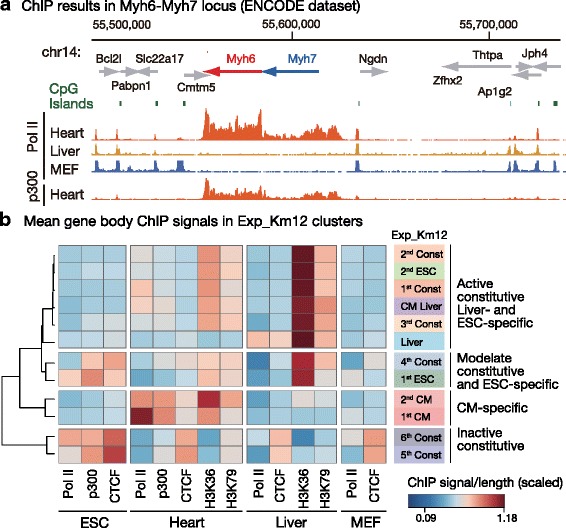
Specific gene-body epigenetic status demarcated cell-type-specific DNA hypomethylated genes in CMs. **a** Landscape of RNA polymerase II (Pol II) and acetyltransferase p300 binding in the *Myh6-Myh7* locus. The gene symbols of proximal genes are shown. **b** Gene-body summary of ChIP signals categorized by Km12 cluster expression. Pol II: RNA polymerase II. MEF: mouse embryonic fibroblasts

As the 5hmC regions in neonatal CMs were marked CM-specific genes while corresponding distributions were broader than the most heavily hypomethylated regions, other factors may also contribute to the identification of 1st and 2nd CM groups in mature CMs. Additionally, to demonstrate whether local molecular interactions are associated with gene-body DNA demethylation in CMs, we examined the distribution of several DNA-binding molecules from the ENCODE/LICR database [29]. First, we found that the *Myh6*-*Myh7* locus shows extremely high accumulations of RNA Pol II and of histone acetylase p300 in both the promoter and GBRs (Fig. 4a). As the observed molecular accumulation was cell-type-specific and overlapped with cell-type-specific hypomethylated areas, we investigated under which category such cell-type-specific molecular interactions in GBRs are established among Exp_Km12 clusters.

In the ENCODE dataset, clustering within Exp_Km12 groups showed that gene-body active histone marks H3K36me3 and H3K79me2 were dominant factors and largely separated active components from inactive components (Fig. 4b). Liver- and ESC-specific genes were found in the same cluster as constitutively active genes while CM-specific genes and constitutively inactive clusters formed two independent branches. The highest accumulations of Pol II were found in the 1st CM-specific gene group, and gene-body Pol II accumulation was also observed in liver- and ESC-specific genes of each cell type at lower concentrations. P300 accumulation in CMs was specific in the 1st CM-specific gene group and in CM-specific genes with lower expression levels (2nd CM) and exhibited patterns most similar to those of 5hmC (Fig. 3a). Weak gene-body CTCF accumulation was observed both in cell-type-specific genes and in the least active constitutive gene groups (5th and 6th Const) of each cell type (Fig. 4b), showing that dynamic binding sites formed in cell-type-specific genes while constitutive binding sites formed in constitutively inactive GBRs (Additional file 1: Figure S7).

Strikingly, although moderate levels of H3K36me3 modification were observed in GBRs of the most active genes of the heart, CM-specific genes with lower expression levels (2nd CM) showed stronger accumulations of H3K36me3 signals (Fig. 4b). Strong gene-body Pol II enrichment roughly overlapped with the 1st CM cluster while H3K36me3 accumulation was suppressed relative to 2nd CM genes. No accumulation of Pol II was observed in inactive hypomethylated constitutive gene regions, showing that hypomethylated GBRs present different characteristics in active and inactive areas.

### Linkages between pol II and gene-body DNA hypomethylation distinguish cell-type-specific genes from HK genes in CMs.

**Fig. 5:**
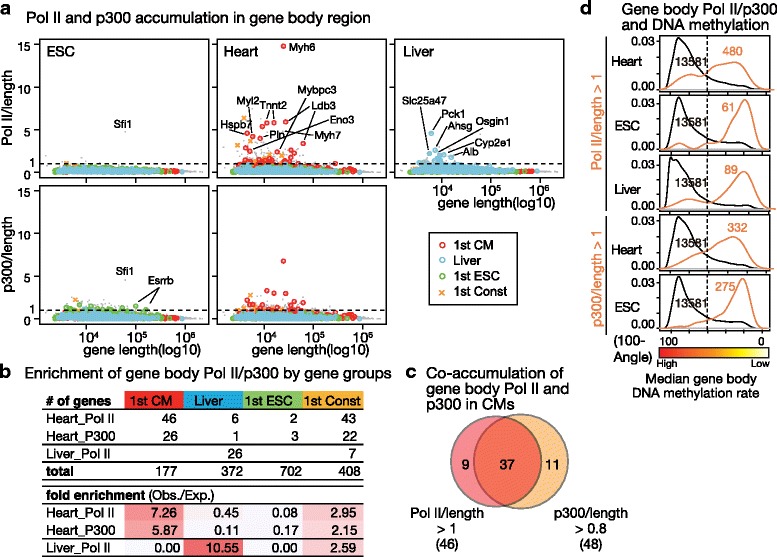
Gene-body Pol II and p300 accumulation was established and accompanied by DNA hypomethylation in adult tissues. **a** Pol II and p300 accumulation in the GBRs. ChIP signal values were normalized by gene length. Gene symbols of representative genes are shown. **b** Number and fold enrichment of Pol II/p300 accumulated genes in corresponding GBRs (signal/length > 1) of four gene groups. **c** Co-localization of Pol II and p300 accumulation in GBRs. **d** Gene-body DNA methylation levels in the Pol II- or p300-accumulated genes (orange lines)

Levels of Pol II and P300 accumulation in GBRs normalized by gene length show an enrichment of CM- and liver-specific genes in corresponding cell types (Fig. 5a and b). As ESCs seldom accumulated Pol II in their GBRs, the formation of such specific epigenetic domains may reflect a specific feature of cellular differentiation. In the heart, the highest and peak net accumulations of both Pol II and p300 were observed at the *Myh6* locus, and Pol II and p300 enriched genes were mostly overlapped (Fig. 5c). Although the hypomethylated GBRs were always a minority, genes with Pol II or p300 were mostly hypomethylated in their GBRs and even in ESCs with few Pol II-enriched genes (Fig. 5d), showing that hypermethylated GBRs heavily sequester the accumulation of Pol II or p300.

**Fig. 6:**
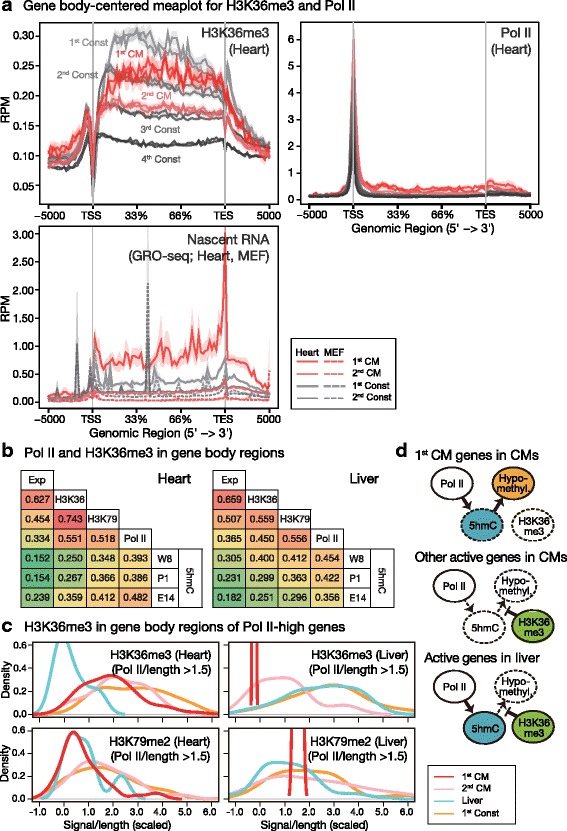
The specific relationship between 5hmC and other marks shapes CM-specific gene-body epigenetic status. **a** Gene-body-centred metaplot of H3K36me3, Pol II and nascent RNA (GRO-seq) in the heart. Read count per million mapped reads (RPM) were mapped according by gene structure and are summarized. Note that the H3K36me3 distribution was lower for 5’ GBRs (near to TSS) of 1st CM genes and that nascent RNA levels were higher in the same gene set than those of 1st Const genes. **b** Correlations between levels of transcription and several gene-body epigenetic modifications in heart and liver tissues. E14: embryonic day 14. **c** Density graph of gene-body H3K36me3 and H3K79me2 levels in Pol II-high genes (Pol II/length > 1.5). Line colours denote groups in Exp_Km12. **d** Models for targeted cell-type-specific gene-body DNA hypomethylation

As H3K36me3 levels and distributions in GBRs are correlated with gene activity [30] and the Pol II rate [31], respectively, we examined the gene body-cantered metaplot of H3K36me3 and Pol II and nascent RNA data from the ENCODE and GEO databases [27, 30], and we found that 1st CM genes are unique in gene body epigenetic modifications. First, the H3K36me3 metaplot shows that gene-body H3K36me3 levels were suppressed in the 1st CM cluster relative to those of the 1st Const gene group (HK genes) and that in contrast to 2nd CM genes, they present similar signal patterns as those of transcriptional level-matched 3rd Const genes. Second, in addition to finding a reduction in H3K36me3 accumulation levels, we found the distribution of H3K36me3 to differ between CM-specific genes (1st CM) and HK genes and especially with a reduction in 5′ end GBRs (Fig. 6a). As slow Pol II elongation tends to shift H3K36me3 distributions towards the 5′ end [31], CM-specific genes (1st CM) appear to be transcribed faster than HK genes. The Pol II metaplot shows that gene-body Pol II levels were uniformly elevated from the 5′ end to the 3′ end (Fig. 6a). In addition, nascent RNA profiling results (GRO-seq) show that active elongating Pol II were highly concentrated in 1st CM genes, showing that high Pol II accumulation in CM-specific genes (1st CM) results from a rapid and active Pol II transcription rate rather than from Pol II pausing or stalling.

The gene-by-gene correlation analysis shows that gene-body H3K36me3 levels served as the best predictor of expression levels in both tissues (Fig. 6b). In contrast, gene-body 5hmC levels in the heart were less correlated with levels of H3K36me3 but were more closely correlated with levels of H3K79me2 and Pol II. These findings differ from those found for liver tissues, for which levels of H3K36me3, H3K79me2 and Pol II were found to be equally correlated with 5hmC levels. H3K79me2 was suppressed in Pol II-high genes in both heart and liver tissues, forging a stronger correlation with H3K36me3 and a potential sequestration of H3K36me3 from Pol II-high genes in the heart (Fig. 6c). Interestingly, the strongest correlation between gene-body 5hmC levels in adult CMs and Pol II or H3K79me2 in the heart was observed in the embryonic stage (Fig. 6b), showing that this link is established at an earlier developmental stage. In contrast, the gene-body 5hmC level measured in the liver showed an increased correlation with Pol II and active histone marks that is presumably the result of 5hmC concentrations found in active genes after birth (Figs. 3a, 6b). Thus, epigenetic differences between constitutive and cell-type-specific genes were found in high Pol II genes in the heart while such differences were less evident in the liver (Fig. 6c).

## Discussion

While CMs rarely divide in adult tissues, the cell division rate was maintained over a week after birth [32]. In contrast, hepatocytes maintained their DNA replication rate even after birth for maintaining their growth and also maintained their regeneration capacities [33]. The different gene-body methylation levels found between CMs and liver cells appear to correspond with the timing of 5hmC accumulation and with gene sets in which 5hmC accumulates in such cell types. Given that gene body 5hmC in hepatocytes was co-localized with general transcriptional activity, 5hmC maintenance is likely driven by transcriptional activity. We found Pol II accumulation in corresponding gene-body regions of adults in regions with transient gene body 5hmC accumulation in earlier stages. Gene-body hypomethylation in CM-specific active genes may occur through further demethylation events via concentrated demethylation activity. Interestingly, the genetic distribution of Pol II was observed in the same region, and H3K36me3 was also lost from CM-specific genes presenting particularly high levels of transcription activity, and therefore, the normal active epigenetic mark involving gene-body methylation may have been compromised by hypomethylation. We examined whether concentrations of 5hmC in CM-specific genes may be affected by gene length, as many CM-specific genes to be transcribed at high levels tend to be longer.

Active transcription generally induces gene-body DNA methylation via phosphorylated Pol II-directed H3K36me3 and subsequent Dnmt3 recruitment [17]. On the other hand, 5hmC, an oxidized derivative of 5mC, is accumulated in the active GBRs of neuronal tissue [21–23], revealing a direct interaction occurring between active transcription and 5hmC. Although the two methylation changes are seemingly paradoxical, the co-localization of H3K36me3 and 5hmC is actually effective in maintaining 5hmC, as the Tet protein requires access to 5mC [34]. This co-localization was observed in all actively transcribed genes of liver cells in our study. As the sequestration of H3K36me3 was observed only in the gene group with high levels of transcription activity in CM specific genes (1st CM), in CM-specific genes with lower expression levels (2nd CM), this demethylation tendency was prevented through the retention of de novo methylation activity involved in H3K36me3, which occurs in active constitutive genes. The cause of such locus-specific imbalance is unknown. In contrast to H3K36me3 sequestration occurring in high Pol II, although H3K79me2 was relatively depleted from high Pol II gene populations in both tissues, the correlation between H3K36me3 and H3K79me2 was found to be higher in the heart, suggesting that the global rearrangement of molecular interactions of epigenetic modifiers among different cell types may be key to establishing the cell-type-specific DNA methylation epigenome (Fig. 6d).

H3K36me3 levels and distributions in GBRs are generally correlated with gene activity [30] and with the Pol II rate [31], respectively. From our H3K36me3 metaplot, H3K36me3 accumulation in CM-specific genes (1st CM) was suppressed and occurred more often on the 3′ end, implying a potentially high Pol II transcription rate (Fig. 6a). This observation does not conflict with high transcriptional levels observed and with high levels of Pol II accumulation observed in the CM-specific genes (1st CM). In the heart, Smyd1 and Smyd2 are presumably the main H3K36 methylases involved; they are highly expressed from the late embryonic stage to 1 week after birth in heart tissues [35]. Interestingly, Smyd1 knockout mice are embryonically lethal at E10 with dilated hearts with abnormally accumulated ECM in heart tissues [36]. Although the CM-specific deletion of Smyd2 is viable, Smyd2 interacts with Pol II and RNA helicase [35], indicating that H3K36 methylases may interact with both transcriptional repressors and translational activators. In our GO analysis, the CM-specific genes with lower expression levels (2nd CM) were enriched with proteinaceous ECM genes (z-value = 7.5642), suggesting that H3K36me3 may suppress the transcription of long genes and especially in CMs. Curiously, ECM expansion in Smyd1-deficient mice may be mediated by the regulation of the translation process, as it does not involve the transcriptional activation of related genes [36], directing our attention to the link between gene body epigenetic modification and translation regulation.

On the other hand, 5hmC may function as a dynamic epigenetic mark. ES cells accumulate under higher 5hmC levels after the M-phase, which can act as a branch point to different cell lineages [37, 38]. Through the elucidation of DNA demethylation interventions of such genomic functions and activities, the role of 5hmC as a key to dynamics of local epigenetic phenomena may be demonstrated. We found that high levels of gene-body Pol II or p300 accumulation are associated with cell-type-specific hypomethylated regions present in an extreme gene population of CMs. Regarding local molecular interactions, 5hmC can serve as a strong recruiter of transcriptional activators as indicated by previous systematic studies [14, 15]. A recent knockdown study of Dnmt3s in human cells shows that 5hmC engages in transcriptional activity in enhancer regions [39]. As 5hmC accumulation in CM-specific genes during the neonatal period resulted in the formation of highly demethylated regions at the adult stage, oxidized derivatives, 5fC and/or 5caC may recruit additional factors to form specific active epigenetic domains. In the case of 5fC, the recruitment of p300 and CTCF [40] and the subsequent activation of Tet by CTCF [41] might establish a sustainable higher structure [42]. Following gene-body p300 modification, a transcriptional elongation activator, Brd4, associates with acetylated histones [43] and plays a role in stress-related heart disease [44]. To elucidate the important role of 5hmC demethylated derivatives, molecular mechanisms shaping the onset of complex molecular interactions must be identified.

Collectively, in CM, 5hmC was accumulated in a gene with comparatively high levels of transcription activity in early stages of development from the balance between gene length and transcription activity. Gene body hypomethylation occurred, and the disturbance of the recruitment of H3K36me3 methylase led to the formation of GBRs presenting high levels of transcriptional elongation activity. We thus predicted that gene length is central to this epigenome formation. While the intronic insertion of exogenous sequences has a relatively weak or neutral effect, heterogeneities in gene lengths in the mammalian genome show that evolutionary gene length expansion is heterogeneous; shorter genes present in extant animals may have experienced strong negative pressure against intronic insertion during evolution [45] while the existence of longer genes suggests not only a functional requirement for long polypeptides but also the existence of advantageous epigenetic support for longer gene transcription. Interestingly, while organisms with relatively small genomes including* Ciona *(approximately 150 Mb) undergo mosaic DNA methylation [46] where only gene regions are methylated, in mammals the entire genome is methylated to form an inhibitory epigenome, and it is natural to adapt several epigenetic marks to ensure the transcription of long genes under such repressive conditions. We found a CM-specific epigenomic mode optimized for efficient long gene expression where cell division was almost arrested and a liver-specific strategy optimized to continue to retain proliferative and regenerative potential with relatively short and uniform gene lengths where cell-type-specific epigenetic marks were not noticeable. As a further example, neuronal cell-type-specific genes exhibit extremes in gene length, and their unique epigenetic mode is implicated by their high gene-body 5hmC levels [21–23, 47] and neuron-specific 5hmC reader, MeCP2 [22]. We propose that the formation of such a cell-type-specific epigenetic system may facilitate genomic evolutionary orchestration whereby the acquisition of sufficient transcripts from a larger and more complex genome is a significant achievement.

## Conclusions

Our results reveal the establishment of a CM-specific gene-body epigenetic state that may enhance transcription through frequent transcriptional elongation. CM-specific epigenetic status was established by the neonatal accumulation of 5hmC in CM-specific genes and from subsequent H3K36me3 accumulation specifically suppressed in the most highly expressed CM-specific genes. Both 5hmC and H3K36me3 accumulation occurred at all active genes of liver cells. Based on differences in gene length found among the cell-type-specific gene groups, we found that differences in epigenetic regulation according to cell type may affect the genomic context. This report is the first to describe dynamic epigenetic states and diverse transcriptional regulation patterns found in GBRs during cell maturation in vivo and corresponding effects on evolutionary genomic rearrangements.

## Methods

### Animals

Eight-month-old C57BL6/J male mice and pregnant C57BL6/J female mice were obtained from Japan SLC, Inc. and CLEA Japan, Inc. All experiments were approved by the Animal Care and Utilization Committee of Keio University School of Medicine, following Institutional Guidelines on Animal Experimentation at Keio University.

### Purification of CMs and cardiac fibroblasts from neonatal and adult mouse hearts

To analyze states of cell-type-specific DNA methylation, CMs were purified from neonatal and adult heart tissues. Neonatal pups (C57BL6/j, 1–3 days old) were sacrificed via cold anaesthesia and cervical dislocation. Their whole hearts were excised, transferred immediately to ice-cold phosphate-buffered saline (PBS; Ca^2+^ and Mg^2+^-free) and washed with chilled PBS. The ventricles were then excised, and the auricles were carefully removed. The tissues were subsequently minced into small pieces in 1× Ads buffer (116 mM NaCl, 20 mM HEPES, 1 mM NaH_2_PO_4_, 5.5 mM glucose, 5.4 mM KCl, and 0.8 mM MgSO_4_, pH 7.35) and washed in dissociation buffer (0.01% collagenase/0.025% trypsin in 1× Ads buffer) with gentle stirring for 5 min at 37 °C. The supernatant was then discarded, and fresh dissociation buffer was added to the samples followed by incubation for 15 min at 37 °C with gentle stirring. The supernatant-containing dispersed cells were then collected into a 50-mL Falcon tube with 2 mL of Dulbecco’s modified Eagle’s medium (DMEM)/F12 medium (1:1; Gibco, Carlsbad, CA, USA) supplemented with 20% horse serum (Gibco). This digestion step was repeated three times. Cell suspensions from each digestion step were then pooled and centrifuged at 1000 rpm for 10 min at 4 °C. To enrich CMs, the cell suspensions were applied to a discontinuous Percoll gradient (1.060/1.086 g/mL) formed in 1× Ads buffer and were then centrifuged at 3000 rpm for 30 min with slow deceleration. The lower layer contained the CMs. The purity (> 95%) of the CMs obtained in this manner was verified using MitoTracker Red and via tetramethylrhodamine methyl ester perchlorate (TMRM Red CMXRos) staining (Molecular Probes) [48]. To isolate the CF population, the upper layer was cultured for 4 days in DMEM/F12 medium supplemented with 20% foetal bovine serum (FBS).

Adult mouse CMs were isolated using a modified version of the collagenase dissociation method. Eight-week-old male mice were Mice were anesthetized with isoflurane gas, and then killed by cervical dislocation. The heart was quickly excised, and the aorta was cannulated for retrograde perfusion in a Langendorff apparatus at a constant flow rate of 3 mL/min at 37 °C. The heart was perfused for 9 to 10 min with isolation buffer (CIB buffer: 120 mM NaCl, 5.4 mM KCl, 1.2 mM MgSO_4_, 1.2 mM NaH_2_PO_4_, 5.6 mM glucose, 5 mM NaHCO_3_, 10 mM HEPES, 50 μM CaCl_2_, 10 mM 2,3-butanedione monoxime [BDM], and 5 mM taurine), followed by digestion for 9 min with collagenase II (1.5 mg/mL; Worthington, Lakewood, NJ, USA) in isolation buffer. After digestion, the soft, flaccid heart was removed and myocytes were suspended in isolation buffer. The CMs were obtained via two mild centrifugation steps, each at 40×*g* for 1 min. The purity (> 95%) of the CMs was verified using a MitoTracker (Molecular Probes) and nuclear staining. To isolate adult CFs, the ventricular tissue was minced and digested as described for the neonatal tissue. The dissociated cells were then plated on plastic dishes [49], and the attached CFs were cultured for 4 days in DMEM/F12 medium containing 10% FBS at a density of 10^4^ cells/cm^2^. The Keio University Ethics Committee for Animal Experiments approved of all of the experiments conducted in this study.

Heart and liver tissues were dissected from foetal (C57BL/6, embryonic day 14), neonatal (1 day after birth) and adult (8 weeks after birth) mice. Mouse ESCs (EB5) were cultured with LIF and serum and were harvested on day 3 of culturing in LIF-supplemented ES medium (15% FBS, 1× GlutaMAX, 1× non-essential amino acids, 1 mM sodium pyruvate, 100 unit/ml penicillin, and 100 μg/ml streptomycin).

### DNA purification

Genomic DNA was separately extracted from isolated cell populations or tissues. Cells or tissues were lysed in sodium dodecyl sulfate (SDS)-containing DNA extraction buffer (100 mM Tris-HCl [pH 8.0], 200 mM NaCl, 50 mM EDTA, and 0.1% SDS) and were treated with proteinase K for 3 h at 55 °C. RNA was subsequently degraded via incubation with RNase A for 1 h at 37 °C, and the lysates were subjected to three rounds of phenol-chloroform-isoamyl alcohol (PCI; Invitrogen) extraction. Finally, the genomic DNA was precipitated with ethanol, was rinsed with 70% ethanol, dried, and was dissolved in TE.

### HELP tagging assay

A HELP tagging assay [50] was performed using genomic DNA from ESCs; foetal, neonatal, and adult CMs; and neonatal and adult CFs. In brief, genomic DNA was digested using either HpaII or MspI. Although these restriction enzymes recognize CCGG sequences, HpaII cleaves only at CCGG sequences in which the central CG dinucleotide is unmethylated. The first Illumina AE adapter is then ligated to the compatible cohesive end that is created, which juxtaposes an EcoP15I site with the HpaII/MspI digestion site and which allows EcoP15I digestion to occur within the flanking DNA sequence. An A-overhang is thereby created, allowing for the ligation of the second Illumina AS adapter. This process enables the generation of both AE-insert-AS and AS-insert-AS products. By performing T7 polymerase-mediated in vitro transcription from a promoter sequence located in the AE adapter, the AE-insert-AS product can be selectively enriched; limited PCR amplification can then be performed to generate a product of a single size for Illumina sequencing. The HELP tagging library sequence data were processed using the HELP pipeline [51]; a brief summary of sequence data is given in Additional file 2: Table S1, and sequences and processed data used are available from the National Center for Biotechnology Information (NCBI) Gene Expression Omnibus (GEO) repository (Accession no.: [GEO: GSE61249]). A HELP analysis was performed once per sample.

The results of the HELP tagging assay were validated via PCR-based bisulfite sequencing whereby raw DNA methylation values were approximated at y = − 1.0113× + 108.33 (R2 = 0.85084) (Additional file 1: Figure S4). In brief, each 300 ng genomic DNA was treated using an EZ DNA Methylation-Gold Kit (Zymo) following the manufacturer’s instructions. PCR was performed using each 10 ng purified converted DNA in a 20 μl PCR reaction mixture using BIOTAQ HS DNA polymerase with 0.8 mM dNTP and 1 mM MgCl2. PCR was performed with 10 min heat activation at 95C followed by 43 cycles of 40 s at 95C, 30 s at 58C, 2 min at 72C, and 10 min at 72C. The size of the DNA was confirmed on agarose gel, size selected and then sub-cloned using a pGEM-T Easy system (Promega). Transformed products in *E. coli* (TOP10, Life Technologies) were amplified by T7 promoter primers, and amplicons were subjected to Sanger sequencing. Sequence data were analysed using QUMA (http://quma.cdb.riken.jp).

### Expression microarray analysis

High-quality RNA was labelled, hybridized, and scanned using the Affymetrix GeneChip microarray platform according to the manufacturer’s instructions. The Affymetrix GeneChip MOE_430A was used for RNA expression profiling. In the present study, the three chips were hybridized with cRNA from neonatal and adult CMs and from neonatal CFs. In brief, 2 μg of total RNA was used to prepare biotinylated cRNA using an in vitro transcription (IVT) labelling kit (Affymetrix) according to the GeneChip Expression Analysis Technical Manual 701025 Rev. 5. The concentration of labelled cRNA was measured using a NanoDrop ND-1000 spectrophotometer, and cRNA quality levels were determined using an Agilent Bioanalyzer (model 2100). Fragmented cRNA was hybridized to the arrays over a period of 16 h at 45 °C. The arrays were then washed and stained in a fluidics station (Affymetrix) and were then scanned using an Affymetrix 3000 GeneScanner. Quality controls for the arrays were included according to the manufacturer’s instructions. To complement the expression data for the liver tissue and ESCs, GSM262266 (liver of a 10-week-old C57BL/6 J male), GSM138289 (liver of an 8-week-old C57BL/6 J female raised on chow diet #36), GSM185513 (R1 ESCs), and GSM198062 (V6.5 ESCs, Genotype 129SvJae x C57BL/6; male; passages 10-15) were considered in the analysis. All of the CEL files were normalized to the RMA and were combined with the epigenome data. Our microarray data are available from the NCBI GEO repository (Accession no.: [GEO: GSE39039]). Two replicates were calculated for each sample to confirm reproducibility.

### 5hmC assay (BGT-mediated 5hmC enrichment (fhM-seal-seq) assay and hMeDIP)

Genomic DNA was divided into fragments of 200 to 500 bp using a Covaris system (Covaris Inc.) and was purified using AMPure (Agencourt). For the BGT assay, a 5mC and 5hmC detection kit (NEB) was employed according to the manufacturer’s instructions with minor modifications. In brief, aliquots of fragmented DNA were incubated overnight with or without 30 units of T4 BGT (NEB) at 37 °C, and some of the aliquots were then digested using 50 units of MspI. The three aliquots were treated with A) T4 BGT + MspI, B) with T4 BGT alone, or C) with MspI alone, and the percentage of 5hmC was calculated as %(A-C)/(B-C).

For the sequencing analysis of pull-down DNA (BGT-seq or hMe_Seal) [52], genomic DNA was fragmented to a length of approximately 300 bp using the Covaris system. Fragmented DNA and UDP-azide-glucose were incubated overnight with T4 BGT (NEB) at 37 °C followed by biotin conjugation. Biotinylated DNA was then purified, captured with streptavidin beads, washed and eluted. The eluted DNA was then purified and employed to construct a DNA library using the ChIP-seq Library Prep Master Mix Set with Q5 (NEB). Library quality was determined using a BioAnalyzer2000, and quantification was performed via qPCR using KAPA Library Quantification Kits (Illumina) followed by Illumina sequencing using the MiSeq and Hiseq2500 platforms; the sequences and processed data are available from the NCBI GEO repository (Accession no.: [GEO: GSE87165]). For paired-end reads, BAM files were generated from PE75 reads mapped by Bowtie2. For single-end reads, BAM files were generated from SE50 reads mapped as pretend paired-end reads of 250 bp in length. From the resulting BAM files, FPKM was calculated for promoter (from TSS-2000 to TSS + 2000) and gene-body (from TSS + 2000 to TES) regions for each Ensemble gene record. To remove replicate gene symbols, gene records of the longest genomic length were chosen for the gene symbol and were used for the calculation. Two independent experiments were performed.

For DNA-IP using a 5hmC antibody, a hydroxymethylated DNA-IP (hMeDIP) assay kit (Active Motif) was employed according to the manufacturer’s instructions with minor modifications. In brief, fragmented DNA was diluted in 10 mM Tris-HCl buffer, denatured at 100 °C for 10 min, and then placed on ice and subjected to IP. A 5hmC antibody (Active Motif) was used for 5hmC DNA enrichment. Enrichment was measured via quantitative PCR. The PCR primers used in these assays are listed in Additional file 2: Table S2. Intact primer target loci were measured in a real-time PCR mixture of a total volume of 20 μL (containing 1X SYBR mix [Applied Biosystems] and 5 μM of each F and R primer) in an ABI Prism 7000 Sequence Detection System (Applied Biosystems). Relative expression levels were calculated based on Gapdh transcript levels. At least three independent PCR experiments were performed.

### Data analysis

The processed HELP tagging data were generated as HELP methylation Angle values with a level of confidence during quantification [51]. Results generated from the eight samples were merged with a cut-off of less than 3 at the calculated degree of confidence, and the HpaII sites for all 8 samples with available data were exclusively chosen as “Analysed HpaII” sites for unbiased comparison. The promoter region was defined as the region spanning 2 kb upstream to 2 kb downstream of the transcription start site (TSS: 4 kb), and the gene-body region was defined as the region spanning 2 kb downstream of the TSS to the transcription termination site.

For the ENCODE/LICR data, “signal” data were calculated as the read frequency per 1 kb per million (FPKM) of the individual gene-body or promoter regions from BAM files (GSE31039, GSE36027). GRO-seq data for heart tissue and MEFs (GSE57926) [53] were also processed after generating BAM files from FASTQ files using Bowtie2 [54]. A gene-body-centred metaplot was generated using ngsplot (https://github.com/shenlab-sinai/ngsplot) [55] from BAM files. All calculations and visualizations were performed using Perl and R statistical software.

Values obtained using different genome-wide analytical methods were processed individually and employed to draw gene-symbol-based comparisons with expression levels determined from microarrays. From the HELP data, median values for both promoters and GBRs were calculated for each RefSeq record. For 5hmC, promoter and gene-body FPKM values were used to draw comparisons. For ChIP-seq data of the ENCODE database, the signal per length was calculated and used to draw comparisons. For the gene-symbol-based comparison of genes to multiple transcript isoforms, the gene record with the longest genomic gene length for each gene symbol was selected for comparison. All calculations were performed using the aforementioned positional definitions of promoter (from TSS-2000 to TSS + 2000) and gene-body (From TSS + 2000 to TES) regions. For our comparison of 5hmC and ChIP data in Km12 clusters, mean FPKM values were normalized using the R “scale” command.

## Additional files

Additional file 1: **Figure S1.** K-means 12 clustering summary. **Figure S2.** Strict gene length restriction in constitutive genes. **Figure S3.** Comparison of gene length and promoter features observed among the 12 cluster populations. **Figure S4.** Cell-type-specific gene body DNA hypomethylation in cardiomyocytes (validation of the HELP tagging method). **Figure S5.** Comparison gene body and promoter DNA methylation patterns. **Figure S6.** Validation of 5hmC enrichment by BGT-qPCR assay. **Figure S7.** Dynamic CTCF binding sites in promoter and gene body regions.

Additional file 2: **Table S1.** Sequence data summary. **Table S2.** PCR primers for bisulfite sequencing and real-time quantification. **Table S3.** Correlation between HELP and MethylC-seq.

## Abbreviations

5caC: 5-carboxylcytosine
5fC: 5-formylcytosine
5hmC: 5-hydroxymethylcytosine
5mC: 5-methylcytosine
BDM: 2,3-butanedione monoxime
BGT: beta-glycosyl transferase
CFs: Cardiac fibroblasts
CGIs: CpG islands
CM: Cardiomyocyte
CpG: C-G dinucleotide
DMEM: Dulbecco’s modified Eagle’s medium
ESCs: Embryonic stem cells
FBS: Foetal bovine serum
GBR: Gene body regions
GEO: Gene Expression Omnibus
H3K27me3: H3K27 trimethylation
H3K36me3: H3K36 trimethylation
H3K79me2: H3K79 dimethylation
HELP: HpaII tiny fragment enrichment by ligation-mediated PCR
HK: Housekeeping
hMeDIP: hydroxymethylated DNA-IP
IVT: in vitro transcription
NCBI: National Center for Biotechnology Information
PBS: Phosphate-buffered saline
PCI: Phenol-chloroform-isoamyl alcohol
Pol II: RNA polymerase II
RMA: Robust multichip average
SDS: Sodium dodecyl sulfate
TDG: Thymine-DNA glycosylase
TMRM Red CMXRos: MitoTracker Red and tetramethylrhodamine methyl ester perchlorate
TSS: Transcription start site
