# Supplementary material for: Selective modulation of local linkages between active transcription and oxidative demethylation activity shapes cardiomyocyte-specific gene-body epigenetic status in mice
Source: BMC Genomics. 2018 May 10;19:349. doi: 10.1186/s12864-018-4752-4 (PMC5946493; doi:10.1186/s12864-018-4752-4)
Supplement: Supplementary file 1 — Figure S1. K-means 12 clustering summary. Figure S2. Strict gene length restriction in constitutive genes. Figure S3. Comparison of gene length and promoter features observed among the 12 cluster populations. Figure S4. Cell-type-specific gene body DNA hypomethylation in cardiomyocytes (validation of the HELP tagging method). Figure S5. Comparison gene body and promoter DNA methylation patterns. Figure S6. Validation of 5hmC enrichment by BGT-qPCR assay. Figure S7. Dynamic CTCF binding sites in promoter and gene body regions. [file 12864_2018_4752_MOESM1_ESM.pdf]

# Supplementary figures

**Supplemental text:**

**Fig. S1: K-means 12 clustering summary.**

A) The proportion of 12 clusters by k-means (Exp_Km12) using the expression profiles of 3 cell types, ESC, CM (cardiomyocytes) and liver tissues. The numbering of clusters, the number of genes used, and the categories used are listed. B) Relative expression levels were plotted. Numbers shown at the upper right denote the number of clusters, and those shown at the bottom left denote the number of genes.

**Fig. S2: Strict gene length restriction in constitutive genes.**

A) Plots of genomic gene length (from TSS to TES, log10) and relative expression levels by expression microarray analysis. Note that the breadth of the gene length of each constitutive gene group grew narrower as expression levels increased. B) Transcription level-matched comparison of cell-type-specific and constitutive gene length. All genes were separated by ranking maximum expression levels for CM (W8), liver (W8) and ESC (Max 1-5) and expression levels for CM (CM 1-5). Note that most (98.3%) of the cell-type-specific genes were of Max 1-3, showing that the longer CM-specific gene subset was observed in all categories with

substantial expression levels. C) Number of Km12 genes in transcription level-matched categories.

**Fig. S3: Comparison of gene length and promoter features observed among the 12 cluster populations.**

A) Genomic gene length and relative expression levels were compared as mean values. Note that in CM, the mean values of the 1st and 2nd CM genes were similar to those of the 1st and 3rd const genes in terms of relative expression levels while they were similar to those of the 5th and 6th const genes in terms of gene length, respectively. B) The proportion of genes with promoter CpG islands and relative expression levels. Note that the proportion of CpG island promoters in the 1st CM was lower than that observed in the 5th const genes.

**Fig. S4: Cell-type-specific gene body DNA hypomethylation in cardiomyocytes (validation of the HELP tagging method).**

A-C) Representative HELP analysis results for cardiomyocyte-specific genes. Orange: promoter regions. (4kb), green: gene body regions. A) *Myh6-Myh7* locus. B) *Myl2* locus. C) *Tnnt2* locus. D) PCR-based locus-specific bisulfite sequencing results. Msp I sites with HELP Angle values are denoted by shaded triangles. E) Correlation of HELP and PCR-based

locus-specific bisulfite sequencing results. F) Comparison of HELP and MethylC-seq. R: correlation coefficiency.

We excluded the possibility that the gene-body hypomethylation of isolated CMs was caused by our CM isolation process, as the DNA methylation levels of these gene-body regions in heart tissues were similar to those of isolated CMs and cardiac fibroblasts (CFs) (Fig. 2C).

**Fig. S5: Comparison gene body and promoter DNA methylation patterns.**

All genes were plotted according to the median angle values of gene body and promoter regions. In all cell types, the majority of genes present hypomethylated promoter regions and hypermethylated gene body regions (high density: red). The population with a hypomethylated gene body with hypomethylated promoters is denoted by dashed rectangles.

**Fig. S6: Validation of 5hmC enrichment by BGT-qPCR assay.**

qPCR validation by BGT assay. The results correspond with DNA-IP and BGT-seq results.

**Fig. S7: Dynamic CTCF binding sites in promoter and gene body regions.**

The number of genes exhibiting promoter or gene body CTCF enrichment (>0.5/bp) was counted. Dynamic CTCF binding was frequently observed in promoters in a heart and/or liver-specific manner while dynamic CTCF binding in shared in promoter and gene body regions was enhanced and specifically in the heart. “PG shared” denotes the number of genes with both promoter and gene body dynamic CTCF binding sites. In heart tissue, promoter and gene body dynamic CTCF binding sites were mostly shared, while roughly half of the gene body dynamic CTCF binding sites in ESC were independent from promoter modification.

Note that Liver-specific and Liver and Heart-common CTCF peaks were most frequently observed in promoter regions (1046 and 1045 peaks were found in promoter regions in total, respectively), while Heart-specific GTCF peaks were observed most frequently in gene body regions (646 peaks were found in the gene body), showing that a dynamic CTCF peak in the heart was more frequently observed in gene body regions than that in other cells.

**Figure S1**

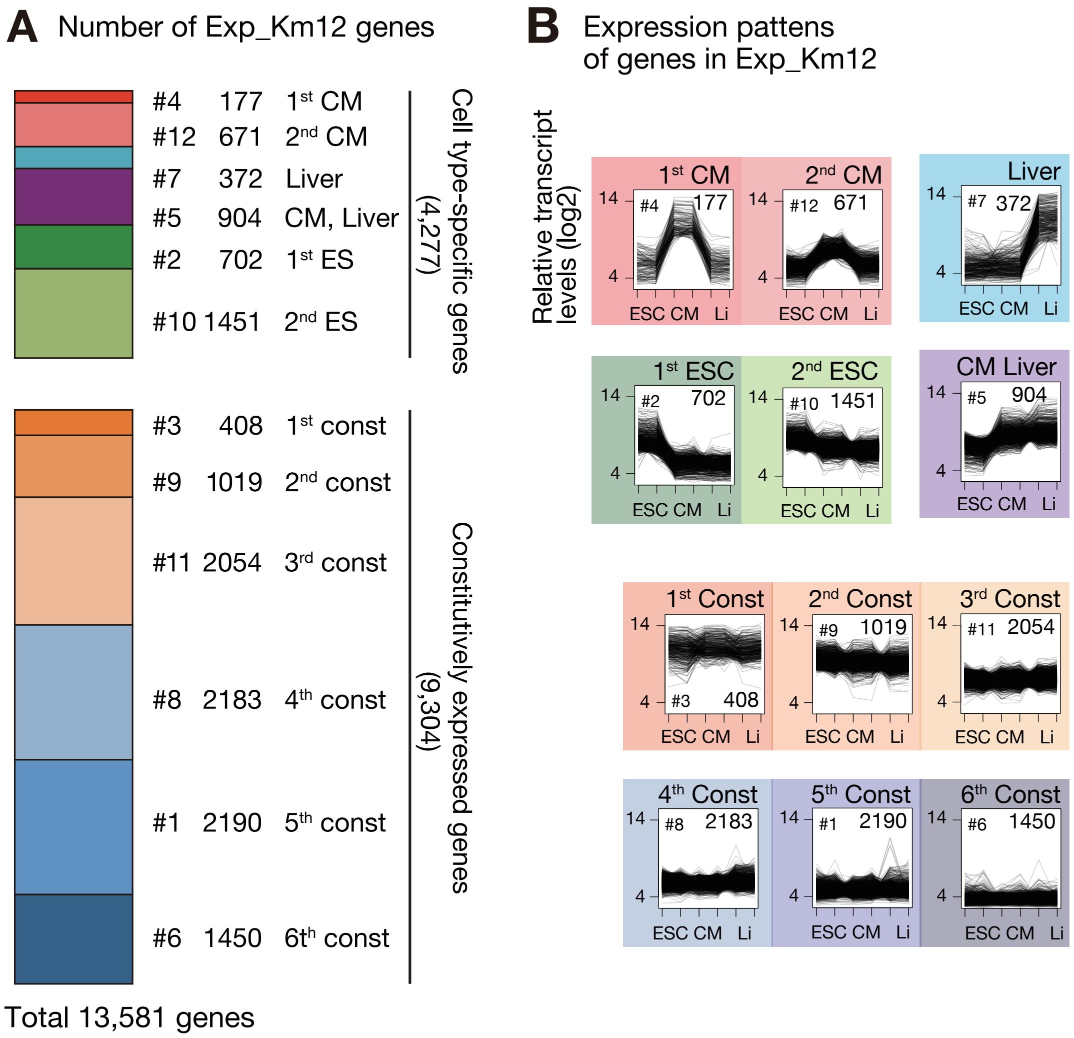
A Number of Exp_Km12 genes
#4 177 1st CM
#12 671 2nd CM
#7 372 Liver
#5 904 CM, Liver
#2 702 1st ES
#10 1451 2nd ES
Cell type-specific genes (4,277)
#3 408 1st const
#9 1019 2nd const
#11 2054 3rd const
#8 2183 4th const
#1 2190 5th const
#6 1450 6th const
Constitutively expressed genes (9,304)
Total 13,581 genes
B Expression pattens of genes in Exp_Km12
Relative transcript levels (log2)
1st CM
#4 177
2nd CM
#12 671
Liver
#7 372
1st ESC
#2 702
2nd ESC
#10 1451
CM Liver
#5 904
1st Const
#3 408
2nd Const
#9 1019
3rd Const
#11 2054
4th Const
#8 2183
5th Const
#1 2190
6th Const
#6 1450
14
4
ESC CM Li

# Figure S2

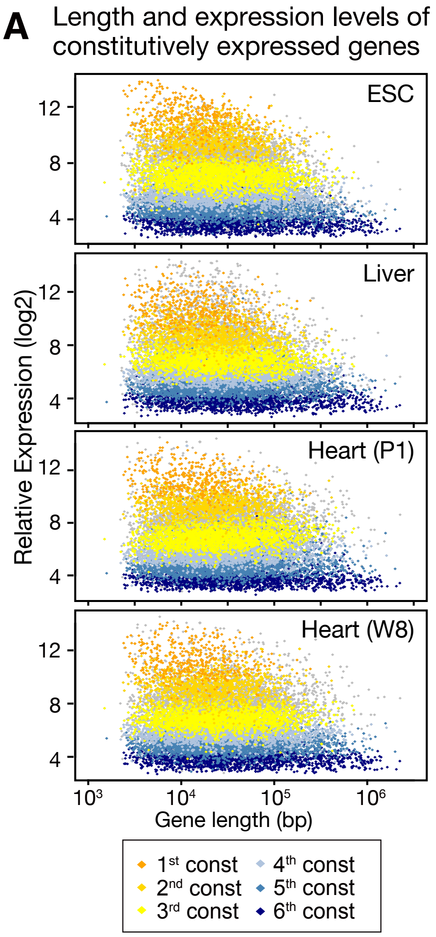


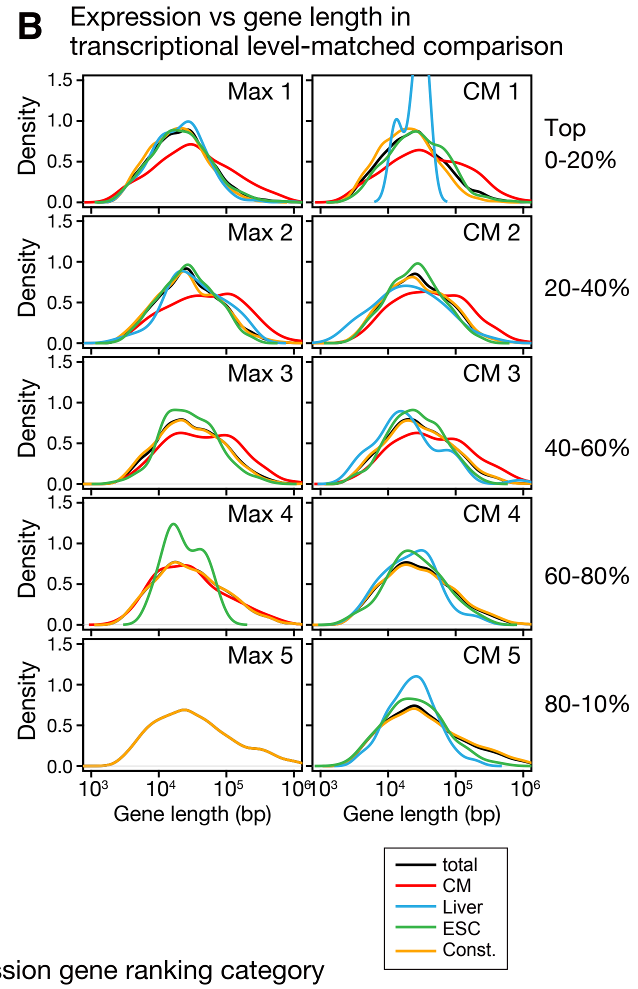


**C** Number of Km12 genes in expression gene ranking category

| | CM | | Liver | CM-Liver | ESC | | Const. | | | | | | |
|---|---|---|---|---|---|---|---|---|---|---|---|---|---|
| | **4** | **12** | **7** | **5** | **2** | **10** | **3** | **9** | **11** | **8** | **1** | **6** | **total** |
| **Max 1** | 174 | 55 | 315 | 393 | 142 | 323 | 408 | 860 | 23 | 11 | 12 | | 2716 |
| **Max 2** | 3 | 262 | 57 | 495 | 261 | 1020 | | 159 | 394 | 41 | 18 | 7 | 2717 |
| **Max 3** | | 301 | | 16 | 279 | 108 | | | 1458 | 429 | 110 | 14 | 2715 |
| **Max 4** | | 53 | | | 20 | | | | 179 | 1671 | 727 | 66 | 2716 |
| **Max 5** | | | | | | | | | | 31 | 1323 | 1363 | 2717 |
| | **4** | **12** | **7** | **5** | **2** | **10** | **3** | **9** | **11** | **8** | **1** | **6** | **total** |
| **CM 1** | 177 | 172 | 4 | 588 | | 371 | 408 | 945 | 52 | | | | 2717 |
| **CM 2** | | 400 | 27 | 290 | 25 | 923 | | 68 | 981 | 1 | | | 2715 |
| **CM 3** | | 99 | 55 | 26 | 224 | 147 | | 5 | 964 | 1077 | 118 | 2 | 2717 |
| **CM 4** | | | 121 | | 289 | 10 | | | 54 | 1062 | 1159 | 21 | 2716 |
| **CM 5** | | | 165 | | 164 | | | 1 | 3 | 43 | 913 | 1427 | 2716 |
| | **CM 1st** | **CM 2nd** | **Liver** | **CM-Liver** | **ESC 1st** | **ESC 2nd** | **Const 1st** | **Const 2nd** | **Const 3rd** | **Const 4th** | **Const 5th** | **Const 6th** | |

# Figure S3

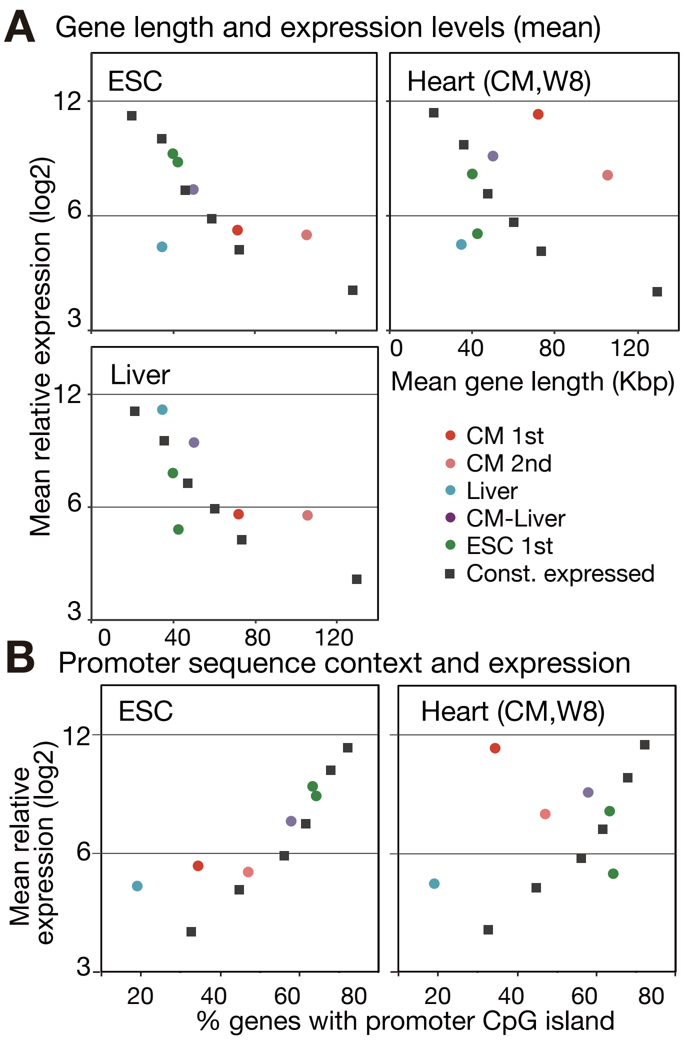

A Gene length and expression levels (mean)
ESC
Heart (CM,W8)
Liver
Mean relative expression (log2)
12
6
3
0
40
80
120
Mean gene length (Kbp)
CM 1st
CM 2nd
Liver
CM-Liver
ESC 1st
Const. expressed
B Promoter sequence context and expression
ESC
Heart (CM,W8)
Mean relative expression (log2)
20
40
60
80
% genes with promoter CpG island

# Figure S4

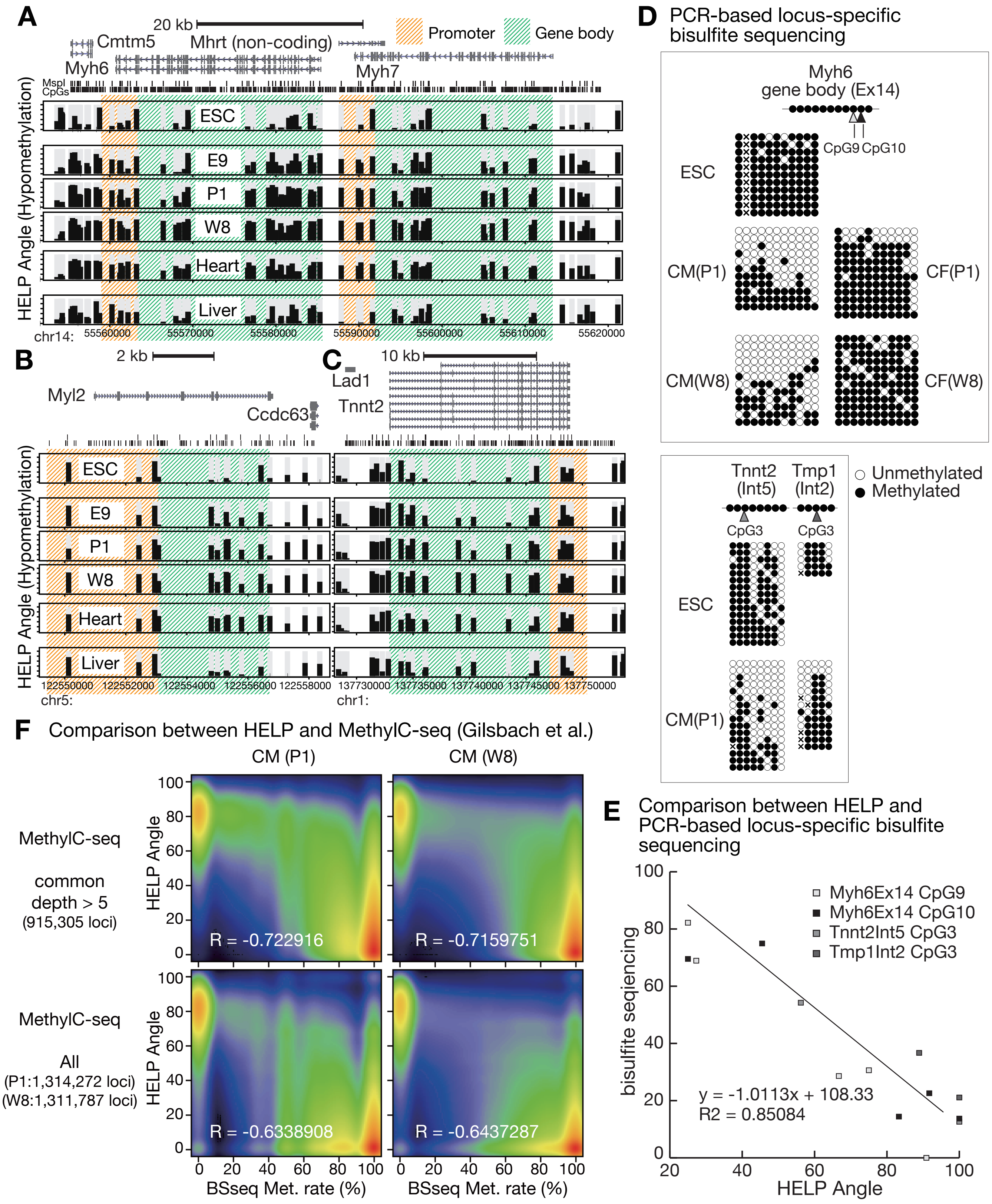

A
20 kb
Promoter
Gene body
Cmtm5
Mhrt (non-coding)
Myh6
Myh7
MspI CpGs
ESC
E9
P1
W8
Heart
Liver
HELP Angle (Hypomethylation)
chr14:
55560000
55570000
55580000
55590000
55600000
55610000
55620000
B
2 kb
Myl2
Ccdc63
C
10 kb
Lad1
Tnnt2
122550000
122552000
122554000
122556000
122558000
chr5:
137730000
137735000
137740000
137745000
137750000
chr1:
D
PCR-based locus-specific bisulfite sequencing
Myh6 gene body (Ex14)
CpG9
CpG10
ESC
CM(P1)
CF(P1)
CM(W8)
CF(W8)
Tnnt2 (Int5)
Tmp1 (Int2)
CpG3
CpG3
Unmethylated
Methylated
ESC
CM(P1)
E
Comparison between HELP and PCR-based locus-specific bisulfite sequencing
Myh6Ex14 CpG9
Myh6Ex14 CpG10
Tnnt2Int5 CpG3
Tmp1Int2 CpG3
y = -1.0113x + 108.33
R2 = 0.85084
bisulfite seqiencing
HELP Angle
F
Comparison between HELP and MethylC-seq (Gilsbach et al.)
CM (P1)
CM (W8)
MethylC-seq
common depth > 5
(915,305 loci)
MethylC-seq
All
(P1:1,314,272 loci)
(W8:1,311,787 loci)
HELP Angle
R = -0.722916
R = -0.7159751
R = -0.6338908
R = -0.6437287
BSseq Met. rate (%)
BSseq Met. rate (%)

**Figure S5**

Relationship between gene body and promoter DNA methylation levels of individual genes (HELP)

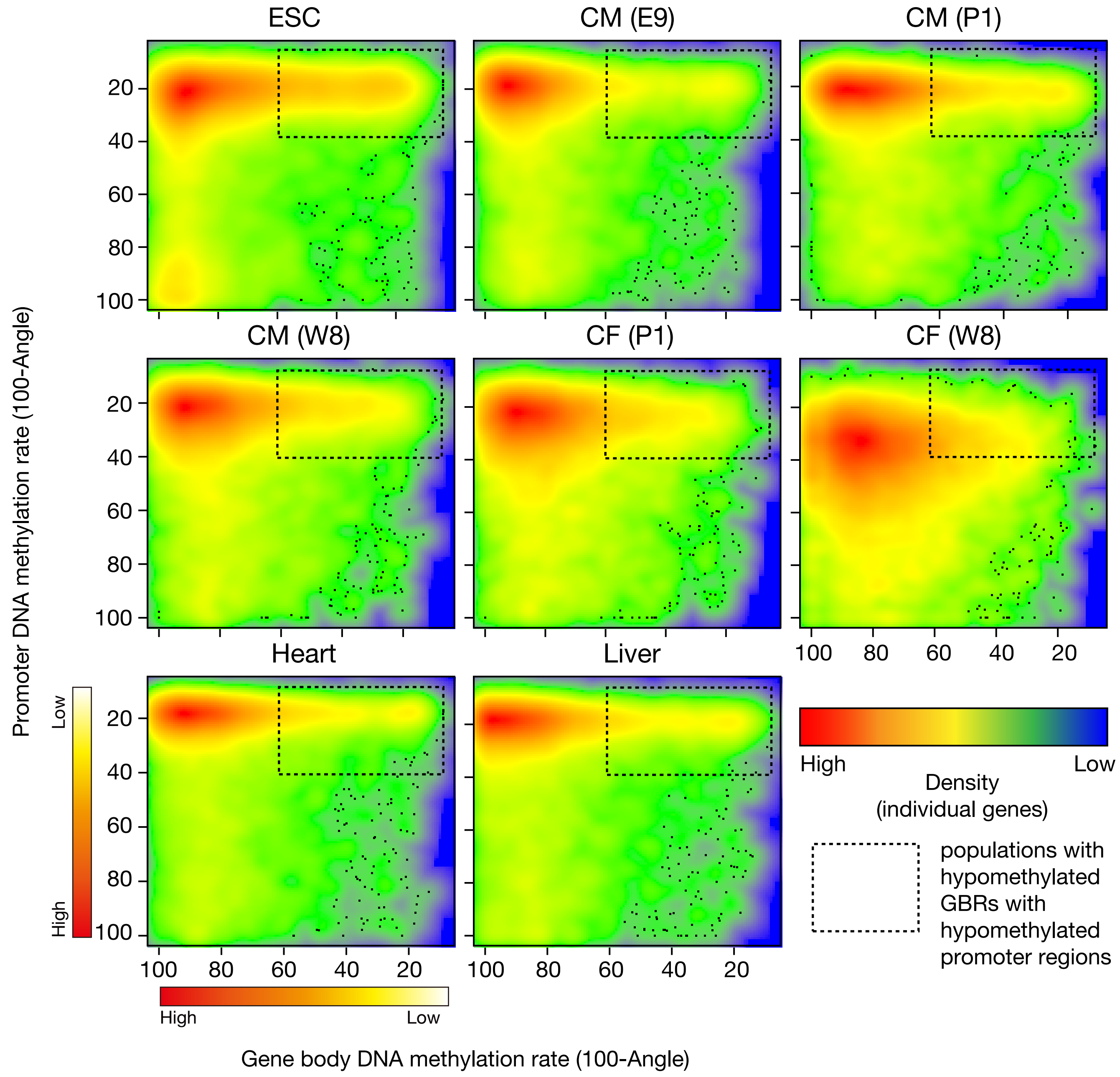

# Figure S6

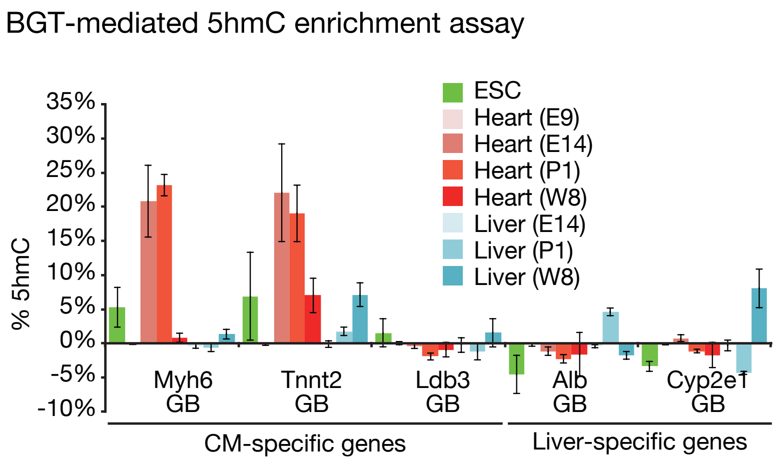
BGT-mediated 5hmC enrichment assay
ESC
Heart (E9)
Heart (E14)
Heart (P1)
Heart (W8)
Liver (E14)
Liver (P1)
Liver (W8)
35%
30%
25%
20%
15%
10%
5%
0%
-5%
-10%
% 5hmC
Myh6 GB
Tnnt2 GB
Ldb3 GB
Alb GB
Cyp2e1 GB
CM-specific genes
Liver-specific genes

# Figure S7

CTCF peak distribution and cell type-specificity

| CTCF peaks | none | Dynamic CTCF binding sites | | | | | | | | | | | | | | Const |
|---|---|---|---|---|---|---|---|---|---|---|---|---|---|---|---|---|
| ES_CTCF+ | 0 | 1 | 0 | 1 | 0 | 1 | 0 | 1 | 0 | 1 | 0 | 1 | 0 | 1 | 0 | 1 |
| Heart_CTCF+ | 0 | 0 | 1 | 1 | 0 | 0 | 1 | 1 | 0 | 0 | 1 | 1 | 0 | 0 | 1 | 1 |
| Liver_CTCF+ | 0 | 0 | 0 | 0 | 1 | 1 | 1 | 1 | 0 | 0 | 0 | 0 | 1 | 1 | 1 | 1 |
| MEF_CTCF+ | 0 | 0 | 0 | 0 | 0 | 0 | 0 | 0 | 1 | 1 | 1 | 1 | 1 | 1 | 1 | 1 |
| **promoter total** | **8259** | **321** | **668** | **90** | **1046** | **107** | **1045** | **331** | **339** | **204** | **95** | **109** | **180** | **132** | **433** | **2733** |
| **gene body total** | **10552** | **524** | **646** | **168** | **221** | **93** | **121** | **178** | **246** | **392** | **115** | **293** | **54** | **199** | **113** | **2177** |
| **PG shared** | **12858** | **331** | **504** | **71** | **394** | **54** | **280** | **97** | **260** | **181** | **70** | **100** | **76** | **79** | **128** | **609** |

Distribution of CTCF binding sites

| promoter | none | Promoter dynamic CTCF binding sites | | | | | | | | | | | | | | Const | #genes |
|---|---|---|---|---|---|---|---|---|---|---|---|---|---|---|---|---|---|
| **1st CM** | 101 | 4 | 47 | 3 | 7 | 0 | 8 | 2 | 6 | 3 | 5 | 3 | 0 | 0 | 7 | 29 | 225 |
| 2nd CM | 409 | 14 | 57 | 13 | 40 | 1 | 36 | 14 | 23 | 8 | 23 | 13 | 7 | 5 | 28 | 149 | 840 |
| **Liver** | 214 | 2 | 6 | 0 | 96 | 16 | 16 | 12 | 0 | 1 | 0 | 0 | 5 | 5 | 4 | 36 | 413 |
| CM, Liver | 363 | 4 | 46 | 4 | 127 | 11 | 152 | 25 | 10 | 1 | 5 | 4 | 22 | 2 | 62 | 258 | 1096 |
| **1st ES** | 476 | 39 | 16 | 13 | 44 | 2 | 35 | 14 | 18 | 7 | 6 | 6 | 9 | 8 | 10 | 117 | 820 |
| 2nd ES | 814 | 12 | 96 | 7 | 141 | 7 | 153 | 41 | 16 | 1 | 5 | 6 | 21 | 11 | 57 | 342 | 1730 |
| **1st const** | 150 | 2 | 32 | 3 | 65 | 3 | 85 | 16 | 1 | 1 | 2 | 0 | 4 | 1 | 15 | 100 | 480 |
| 2nd const | 517 | 0 | 64 | 2 | 113 | 3 | 137 | 37 | 9 | 2 | 7 | 2 | 18 | 5 | 41 | 235 | 1192 |
| 3rd const | 1027 | 31 | 105 | 12 | 153 | 12 | 206 | 84 | 47 | 21 | 8 | 11 | 32 | 20 | 101 | 556 | 2577 |
| 4th const | 1307 | 59 | 103 | 21 | 174 | 22 | 133 | 48 | 68 | 38 | 17 | 19 | 36 | 29 | 64 | 456 | 2426 |
| 5th const | 1589 | 99 | 71 | 10 | 68 | 24 | 61 | 28 | 85 | 77 | 14 | 34 | 20 | 31 | 35 | 331 | 2594 |
| **6th const** | 1292 | 55 | 25 | 2 | 18 | 6 | 23 | 10 | 56 | 44 | 3 | 11 | 6 | 15 | 9 | 124 | 1699 |
| **total** | **8259** | **321** | **668** | **90** | **1046** | **107** | **1045** | **331** | **339** | **204** | **95** | **109** | **180** | **132** | **433** | **2733** | 16092 |

| gene body | none | Gene body dynamic CTCF binding sites | | | | | | | | | | | | | | Const | #genes |
|---|---|---|---|---|---|---|---|---|---|---|---|---|---|---|---|---|---|
| **1st CM** | 119 | 5 | 28 | 5 | 0 | 0 | 0 | 1 | 5 | 2 | 8 | 5 | 1 | 1 | 1 | 44 | 225 |
| 2nd CM | 536 | 17 | 60 | 4 | 3 | 2 | 5 | 12 | 5 | 15 | 10 | 25 | 0 | 5 | 14 | 127 | 840 |
| **Liver 1st** | 252 | 3 | 3 | 0 | 69 | 14 | 6 | 10 | 0 | 1 | 0 | 0 | 4 | 4 | 5 | 42 | 413 |
| CM, Liver | 601 | 16 | 53 | 7 | 36 | 7 | 26 | 19 | 20 | 17 | 6 | 8 | 12 | 22 | 29 | 217 | 1096 |
| **1st ES** | 627 | 44 | 21 | 6 | 3 | 3 | 3 | 4 | 10 | 26 | 3 | 14 | 0 | 9 | 0 | 47 | 820 |
| 2nd ES | 1158 | 46 | 78 | 28 | 15 | 6 | 10 | 23 | 27 | 48 | 12 | 30 | 4 | 19 | 8 | 218 | 1730 |
| **1st const** | 317 | 6 | 29 | 4 | 21 | 7 | 15 | 10 | 4 | 1 | 2 | 3 | 4 | 3 | 9 | 45 | 480 |
| 2nd const | 821 | 22 | 50 | 14 | 14 | 4 | 8 | 21 | 27 | 10 | 15 | 11 | 4 | 12 | 5 | 154 | 1192 |
| 3rd const | 1351 | 99 | 104 | 31 | 21 | 16 | 22 | 19 | 57 | 78 | 19 | 59 | 15 | 54 | 21 | 460 | 2577 |
| 4th const | 1549 | 102 | 115 | 24 | 28 | 18 | 15 | 25 | 49 | 84 | 25 | 66 | 8 | 41 | 9 | 436 | 2426 |
| 5th const | 1741 | 129 | 67 | 33 | 10 | 14 | 10 | 28 | 36 | 90 | 13 | 59 | 2 | 23 | 11 | 311 | 2594 |
| **6th const** | 1480 | 35 | 38 | 12 | 1 | 2 | 1 | 6 | 6 | 20 | 2 | 13 | 0 | 6 | 1 | 76 | 1699 |
| **total** | **10552** | **524** | **646** | **168** | **221** | **93** | **121** | **178** | **246** | **392** | **115** | **293** | **54** | **199** | **113** | **2177** | 16092 |
