# Supplementary material for: Selective modulation of local linkages between active transcription and oxidative demethylation activity shapes cardiomyocyte-specific gene-body epigenetic status in mice
Source: BMC Genomics. 2018 May 10;19:349. doi: 10.1186/s12864-018-4752-4 (PMC5946493; doi:10.1186/s12864-018-4752-4)
Supplement: Supplementary file 2 — Table S1. Sequence data summary. Table S2. PCR primers for bisulfite sequencing and real-time quantification. Table S3. Correlation between HELP and MethylC-seq. [file 12864_2018_4752_MOESM2_ESM.pdf]

Supplementary tables

Table. S1: Sequence data summary.

1) Expression microarray data.

2) HELP tagging data.

3) 5hmC-seq data.

Table. S2: PCR primers for bisulfite sequencing and real-time quantification.

1) Primers for bisulfite sequencing.

2) Genomic PCR primers for real-time PCR quantification.

Table. S3: Correlation between HELP and MethylC-seq.

The bisulfite sequencing method generates two DNA methylation values for one CpG binucleotide. As Hpa II restriction can be affected by the methylation of either C, the higher DNA methylation value of the two corresponds to the Hpa II restriction rate (=HELP Angle value).

Table. S1: Sequence data summary.

1) Expression microarray data.

GSE39039

platform: GPL1261 [Mouse430_2] Affymetrix Mouse Genome 430 2.0 Array

GSM954457	**P1CM_rep1**
GSM1487544	**P1CM_rep2**
GSM954458	**W8CM_rep1**
GSM1487546	**W8CM_rep2**
GSM185513	**ESC (R1)**
GSM198062	**ESC (129SvJae x C57BL/6)**
GSM262266	**Liver (10W,C57BL/6J)**
GSM138289	**Liver (8W,C57BL/6J)**

2) HELP tagging data.

GSE61249

genome: mm9

platform: GPL11002 Illumina Genome Analyzer IIx (Mus musculus)

GEO acssesion number	sample ID	reads with linker	reads aligned(1mismatch)	%mapped	aligned_unique	%alined (unique)
GSM1500756	**ESC**	28,920,141	16,083,481	55.6%	15,618,937	54.0%
GSM1500757	**E9CM**	29,528,483	20,408,729	69.1%	19,903,481	67.4%
GSM1500758	**P1CM**	31,020,569	23,079,456	74.4%	22,455,433	72.4%
GSM1500759	**W8CM**	26,501,570	20,285,250	76.5%	19,180,598	72.4%
GSM1500760	**P1CF**	26,022,007	19,904,906	76.5%	19,402,854	74.6%
GSM1500761	**W8CF**	24,252,745	8,577,115	35.4%	8,370,249	34.5%
GSM1500762	**Heart**	30,963,603	23,536,990	76.0%	22,073,566	71.3%
GSM1500763	**Liver**	26,501,903	20,738,211	78.3%	19,848,560	74.9%

3) 5hmC-seq data.

GSE87165

platform: GPL16417 Illumina MiSeq (Mus musculus) Paired-end

GEO acssesion number	sample ID	read count	alined read count	%alined
GSM2323813	**ESC rep1**	2,832,913	2,683,461	94.7%
GSM2323814	**ESC rep2**	5,398,539	5,078,015	94.1%
GSM2323815	**Heart (P1) rep1**	1,593,848	1,539,856	96.6%
GSM2323816	**Heart (P1) rep2**	1,268,250	1,231,221	97.1%
GSM2323817	**Liver (P1) rep1**	1,262,076	1,213,541	96.2%
GSM2323818	**Liver (P1) rep2**	4,402,253	4,216,392	95.8%

platform: GPL17021 Illumina HiSeq 2500 (Mus musculus) Single-end

GEO acssesion number	sample ID	read count	alined read count	%alined*
GSM2323819	**Heart (E14) rep2**	7,701,208	7,929,426	103.0%
GSM2323820	**Liver (E14) rep1**	15,414,001	15,531,234	100.8%
GSM2323821	**Heart (W8) rep1**	7,181,444	7,257,186	101.1%
GSM2323822	**Heart (W8) rep2**	316,846	326,186	102.9%
GSM2323823	**Liver (W8) rep1**	8,811,203	8,885,883	100.8%
GSM2323824	**Liver (W8) rep2**	8,050,929	8,106,002	100.7%

Table. S2: PCR primers for bisulfite sequencing and real-time quantification.

1) Primers for bisulfite sequencing.

Gene	position	Forward primer	Reverse primer	chrom (mm9)	start (mm9)	end (mm9)	product length	tid	MspI	primer name	category
Myh6	Ex14	GAGGAGAGTTAGGGGTTATATGAATTTT	ATATCTAAACCAACACCCTTCCCAA	chr14	55576287	55576848	562			Myh6_Ex14_bi	CM-specific gene
Tnnt2	Int5	GGTTTTGAAGGTAGAGGTTTGTGAG	ACTCCCTATCCTAATAACCCCACTC	chr1	137,743,360	137,743,817	458			Tnnt2_Int5_bi	CM-specific gene
Tmp1	Int2	AGGAGGAGTATTAGGGGAAGTTATGG	ATTCCTACCCCTTTACACAAACCAC	chr9	66,886,681	66,887,248	568			Tmp1_Int2_bi	CM-specific gene

2) Genomic PCR primers for real-time PCR quantification.

Gene	position	Forward primer	Reverse primer	chrom (mm9)	start (mm9)	end (mm9)	product length	tid	MspI	primer name	category
Myh6	PRO	GGTGTGAGACGCTCCTGTCT	GTTTGCCCATGAAAGGTCTG	chr14	55585578	55585704	127		**Y**	Myh6_PRO_QC_F	CM-specific gene
Myh6	GB	TACCCCCAGACGATCATGTAA	GAAAGGCTCATCCTTCCAAAC	chr14	55576469	55576636	168	451454	**Y**	Myh6_Ex14_QC_F	CM-specific gene
Tnnt2	GB	ACAGGAGTGCATGTGTGCAT	GTGAAGTCAAGGCGACGTTC	chr1	137743404	137743524	121	71051	**Y**	MO_Tnnt2_Int6_QC_F	CM-specific gene
Ldb3	GB	CCAGGTTAGTGGCTGCAAAA	TATGCAGAGGCCAGAGGCTA	chr14	35383639	35383774	136	440039	**Y**	MO_Ldb3_Int4_QC_F	CM-specific gene
Ahgh	GB	CAAGACGCTCCCTGAATGAG	GGTGTGCATCACCCTGAACT	chr16	22894795	22894933	139		**Y**	Ahsg_Int2_QC	Liver-specific
Alb	GB	TGTGCTGGGACATCTCCTTT	AGCACTCCCTTCTGGCAAAT	chr5	90894534	90894668	135		**Y2**	Alb_Int5_QC	Liver-specific
Cyp2e1	GB	TGACAAGGACCTGTGGGAAG	CACAGGAAGAACACCGGAGA	chr7	147952756	147952977	222		**Y2**	Cyp2e1_Int2_QC	Liver-specific
Pck1	GB	ACTAACCCCGAAGGCAAGAA	GACTCACCTTGGGCATCAAA	chr2	172981451	172981608	158		**Y**	Pck1_Ex4_QC	Liver-specific

Table. S3: Correlation between HELP and MethylC-seq.

a) depth >10, 222,489 loci (20.3%)

bisulfite_H	rep.	HELP Angle	rep.	C.C
P1CM	1	P1CM	1	**-0.744**
P1CM	2	P1CM	1	**-0.743**
P1CM	3	P1CM	1	**-0.746**

bisulfite_H	rep.	HELP Angle	rep.	C.C
W8CM	1	W8CM	1	**-0.723**
W8CM	2	W8CM	1	**-0.729**
W8CM	3	W8CM	1	**-0.729**

bisulfite_H	rep.	HELP Angle	rep.	C.C
P1CM	1	Liver	1	**-0.631**
P1CM	2	Liver	1	**-0.630**
P1CM	3	Liver	1	**-0.632**

bisulfite_H	rep.	HELP Angle	rep.	C.C
W8CM	1	Liver	1	**-0.614**
W8CM	2	Liver	1	**-0.621**
W8CM	3	Liver	1	**-0.621**

b) depth >5, 915,305 loci (83.3%)

bisulfite_H	rep.	HELP Angle	rep.	C.C
P1CM	1	P1CM	1	**-0.723**
P1CM	2	P1CM	1	**-0.722**
P1CM	3	P1CM	1	**-0.727**

bisulfite_H	rep.	HELP Angle	rep.	C.C
W8CM	1	W8CM	1	**-0.716**
W8CM	2	W8CM	1	**-0.718**
W8CM	3	W8CM	1	**-0.718**

bisulfite_H	rep.	HELP Angle	rep.	C.C
P1CM	1	Liver	1	**-0.651**
P1CM	2	Liver	1	**-0.650**
P1CM	3	Liver	1	**-0.654**

bisulfite_H	rep.	HELP Angle	rep.	C.C
W8CM	1	Liver	1	**-0.644**
W8CM	2	Liver	1	**-0.646**
W8CM	3	Liver	1	**-0.646**

***bisulfite_H:** bisulfite sequencing method provides two DNA methylation values for one CpG binucleotide. Since Hpa II restriction can be affected by methylation of either C, the higher DNA methylation value of two would be correspond to Hpa II resctriction rate (=HELP Angle value).
